# Focal adhesion kinase–related pathways may be suppressed by metformin in vascular smooth muscle cells in high glucose conditions

**DOI:** 10.1002/edm2.351

**Published:** 2022-05-28

**Authors:** Ali Akbar Soleimani, Ghasem Ghasmpour, Asghar Mohammadi, Masoomeh Gholizadeh, Borhan Rahimi Abkenar, Mohammad Najafi

**Affiliations:** ^1^ Clinical Biochemistry Department, Faculty of Medicine Iran University of Medical Sciences Tehran Iran; ^2^ Clinical Biochemistry Department, Faculty of Medicine Tarbiat Mdares University Tehran Iran; ^3^ Clinical Biochemistry Department, Faculty of Medicine Golestan University of Medical Sciences Gorgan Golestan Iran; ^4^ Cellular and Molecular Research Center Iran University of Medical Sciences Tehran Iran

**Keywords:** FAK, high glucose, metformin, migration, VSMCs

## Abstract

**Introduction:**

Cardiovascular diseases are known as one of the important causes of death in patients with diabetes mellitus. Metformin is used as an oral medication for reducing blood sugar. In this study, the effects of metformin were investigated on the FAK gene expression levels, pFAK protein values, cell viability and migration rate of VSMCs in high glucose conditions.

**Materials and methods:**

The FAK gene expression levels and pFAK protein values were evaluated in VSMCs treated with different doses of metformin (1, 5 and 7 mM), based on cell viability using RT‐qPCR, western blotting and MTT techniques. The cellular migration was evaluated by scratch assay.

**Results:**

The FAK gene expression levels reduced significantly in metformin‐treated VSMCs at 24 h and 48 h periods (*p* < .0008 and *p* < .0001, respectively). The pFAK protein values reduced significantly at 24 h (5 mM and 7 mM metformin doses) and 48 h periods (*p* < .001). In agreement with pFAK protein values, cellular migration reduced significantly at 24 h and 48 h periods (*p* < .001).

**Conclusion:**

The results showed that metformin may suppress the proliferation and migration of VSMCs via FAK‐related pathways and may retard the progression of vessel stenosis in diabetes.

## Graphical abstract

The results showed that metformin may suppress the VSMC proliferation and migration via the decrease of FAK in focal adhesion pathway. Furthermore, data suggested that metformin may retard atherosclerosis process in patients of diabetes.

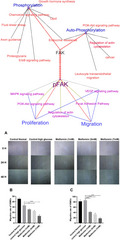

## 1 INTRODUCTION

Cardiovascular diseases account for approximately 65 percent of deaths in patients with diabetes mellitus.^1^ It is well known that the pathogenesis of atherosclerosis is related to the proliferation and migration of vascular smooth muscle cells (VSMCs) within the media layer of arteries, so that these events promote atherogenic plaques led to vessel stenosis and restenosis. Molecular mechanisms behind VSMC proliferation and migration remain unclear.^2^, ^3^ Hyperglycemia, however, is not the only factor involved in the progression of cardiovascular diseases in diabetics but it is involved in several pathways including the activation of protein kinase C, the formation of advanced glycation end‐products (AGEs) and the stimulation of lipoxygenase synthesis pathway.^4^

Metformin is an oral medication used commonly for patients with type 2 diabetes and other diseases related to insulin resistance.^5^, ^6^, ^7^ Moreover, metformin affects the liver, skeletal muscle and adipose tissues via decreasing oxidative stress, improving insulin sensitivity, blocking gluconeogenesis and promoting glucose uptake and consumption.^5^, ^6^, ^7^, ^8^ Metformin also inhibits VSMC function by targeting the AMPK signalling pathway, which may retard the process of atherosclerosis in diabetes.^9^

**FIGURE 1:**
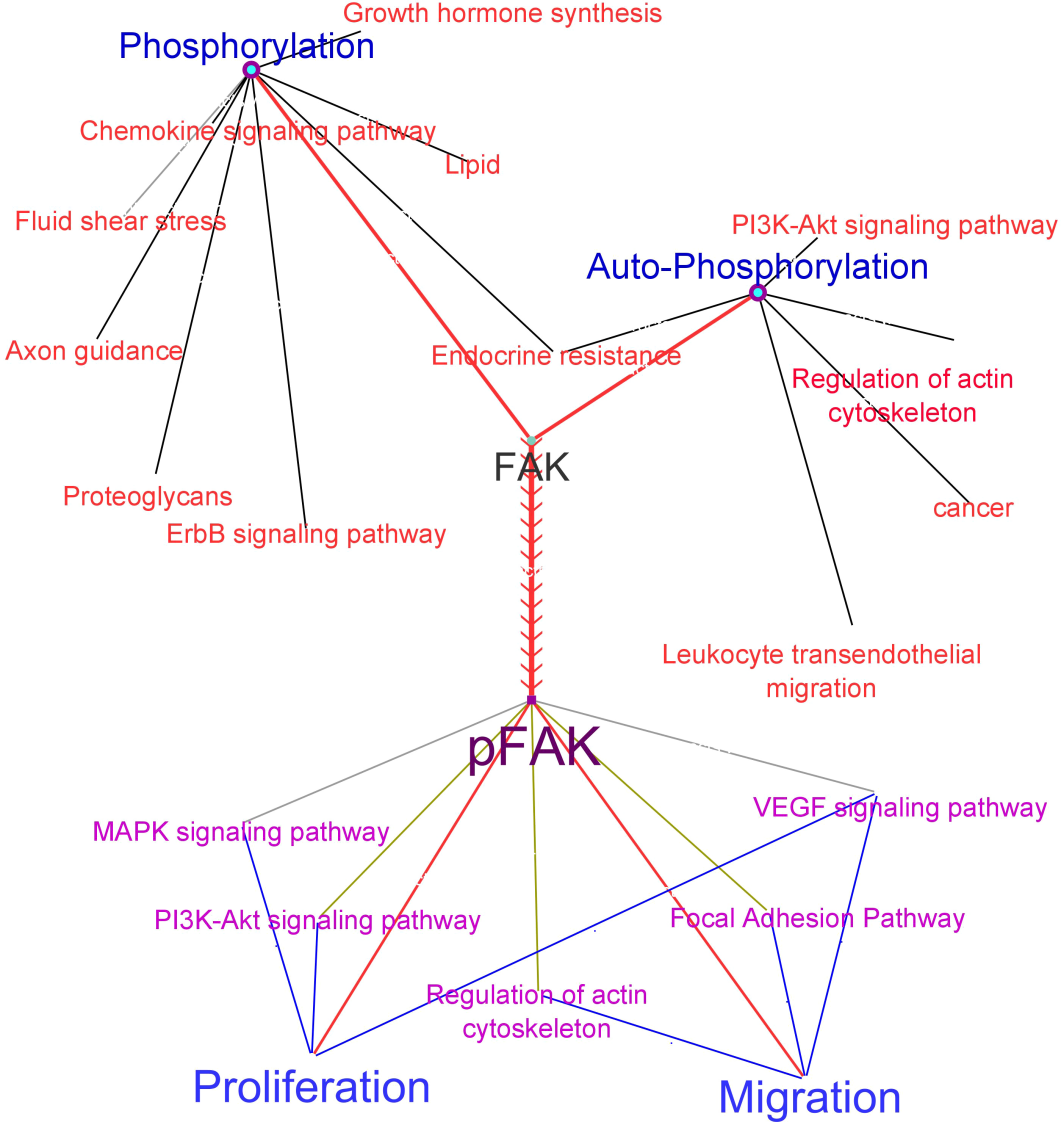
Cellular proliferation and migration via FAK/pFAK axis. The FAK is phosphorylated by kinase‐followed signalling pathways or autophosphorylated by the cellular membrane receptors. Then, pFAK transduces the signals into the downstream proteins directly or by other the cross‐talked signalling pathways led to the cellular proliferation and migration events

FAK is a tyrosine kinase that regulates cell proliferation and migration through some signalling pathways.^10^ Several agents such as inflammatory factors, cytokines and growth factors activate the focal adhesion kinase.^11^ Since pFAK (phosphorylated FAK) was considered as a central protein to transduce a signal into the cell nucleus in the cellular signalling pathways (Figure 1), thus in this study, the effects of metformin were investigated on the changes in FAK gene expression levels, pFAK protein values, cell viability and migration rate of VSMCs in high glucose conditions.

## 2 METHODS AND MATERIALS

### 2.1 Cell culture

Vascular smooth muscle cells were obtained from the National Cell Bank (NCBI code, C591), Pasteur Institute (Tehran, Iran) and were cultured in DMEM (Gibco, NY, USA) containing fetal bovine serum (FBS) 10% and penicillin–streptomycin 1% (Bioidea Company). The cell groups (control, high glucose (final concentration, 25 mM), and metformin and high glucose (1, 5 and 7 mM)) were cultured (70% confluency) and treated for 24 and 48 h.

### 2.2 Cell viability

Cell viability was carried out using the MTT (methyl thiazol tetrazolium, Sigma‐Aldrich) method. Briefly, 4000 cells per well were seeded in the 96‐well plate and were incubated with different doses of metformin for 24 and 48 h periods. Then, the medium was discarded and the seeded cells were incubated with MTT solution (0.5 mg/ml; Sigma‐Aldrich) for 4 h at 37°C. After removing the MTT solution, 200 μl of DMSO (Dimethyl sulfoxide) was added to dissolve the formed crystals, and finally, the plate was shaken for 15 min at room temperature. Afterwards, optical density was measured at 570 nm wavelength.

### 2.3 Real‐time qPCR technique

Total RNA was extracted from VSMCs by GeneAll‐Hybrid‐R RNA purification kit (Seoul, South Korea). cDNA synthesis was performed according to the SMOBIO kit protocol (Hsinchu, Taiwan). SYBR Green PCR Master Mix (Amplicon Denmark) was used for real‐time RT‐PCR reaction. The gene expression level was normalized by GAPDH gene. The reactions for all genes were carried out in volumes of 15 μl. The temperature cycles (45 cycles) were performed at 95°C (10 s) and 60°C (45 s). Primer‐BLAST (NCBI.nlm.nih.gov/tools/primer‐blast) and OligoCalc servers were used to design gene primers (GAPDH, F‐CATGAGAAGTATGACAACAGCCT, R‐AGTCCTTCCACGATACCAAAGT; FAK, F‐CATGCCCTCAACCAGGGATT R‐CACGCTGTCCGAAGTACAGT). The gene expression changes were calculated by the 2^−ΔΔCT^ formula.

### 2.4 Western blotting technique

RIPA buffer (Santa Cruz Biotechnology)‐containing protease inhibitors (protease inhibitor cocktail and phenylmethylsulfonyl fluoride [PMSF]) were added to the cell pellet and were centrifuged at 13,000 *g* (4°C, 20 min) to extract the total protein. The protein value was measured by the Lowry method (Sigma). 10 μl of protein was run and electrophoresed on sodium dodecyl sulfate–polyacrylamide gel (12%) for 45 min (90 V). Then, the protein bands were transferred to PVDF (polyvinylidene difluoride) membrane (Merck Millipore) for 60 min (90 V). The PVDF membrane was blocked with 4% w/v fat‐free milk (Nonfat Dry Milk #9999; Cell Signalling Technology) for 60 min and was incubated with primary FAK antibody (CAT.#:3283 s; 1:1000 v/v; Cell Signalling Technology) overnight at 4°C. Then, the PVDF membrane was washed in tris‐buffered saline with 0.1 percent tween ® 20 (TBST) and was incubated with secondary antibody (CAT.#:7074 s; 1:10000 v/v; Cell Signalling Technology) 60 min at room temperature. Finally, the membrane was exposed to enhanced chemiluminescence (ECL) reagent (RPN2235; Amersham Biosciences). Beta‐actin antibody (CAT.#:4967 s; 1:1000 v/v; Cell Signalling Technology) was used to normalize the protein values. The band densities were identified using Image‐J software.

### 2.5 Scratch assay

To investigate cellular migration, a scratch assay test was performed. The VSMC cells were first cultured in 12‐well plates and then were scratched with a pipette tip. After being washed with PBS (Sigma‐Aldrich), the cells were incubated for 24 h and 48 h periods in media containing metformin (1, 5 and 7 mM). An inverted microscope was used to obtain images of the wounded area (OPTIKA). The analysis of images was accomplished by Image‐J software.

### 2.6 Statistical analysis

The data were analysed by Graphpad Prism (Version 8.0.3). Initially, data distribution was evaluated by the Kolmogorov–Simonov test. Then, data were compared using the ANOVA test. *p* < .05 was the subject to be considered significant.

### 2.7 Ethical approval

This experiment was approved by the Ethics Committee of Iran university of medical sciences with the following ethical code: IR.IUMS.FMD.REC.1400.182.

## 3 RESULTS

### 3.1 Cell viability

**FIGURE 2:**
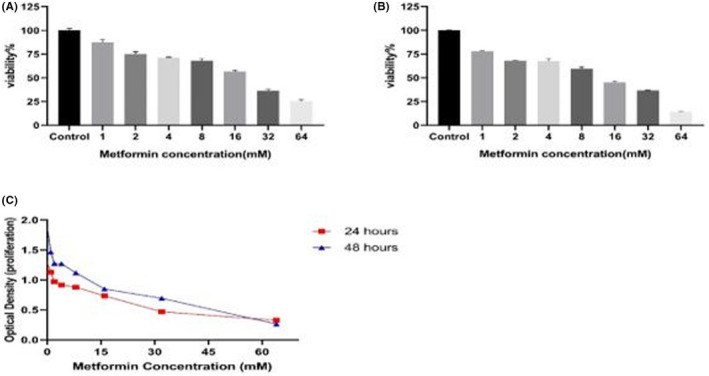
VSMC viability. The VSMCs were treated with different metformin values (0–64 mM). Cellular viabilities were studied at periods of 24 h (A) and 48 h (B). (C) The cell viability is based on optical density. Data are repeated three times (*n* = 3) and are presented in mean ± SD

The viability of vascular smooth muscle cells was determined after the treatment with different values of metformin. In comparison with the control group in both periods of 24 h and 48 h, the treatment with metformin reduced VSMC survival and proliferation in a dose‐dependent manner (Figure 2).

### 3.2 FAK gene expression levels

**FIGURE 3:**
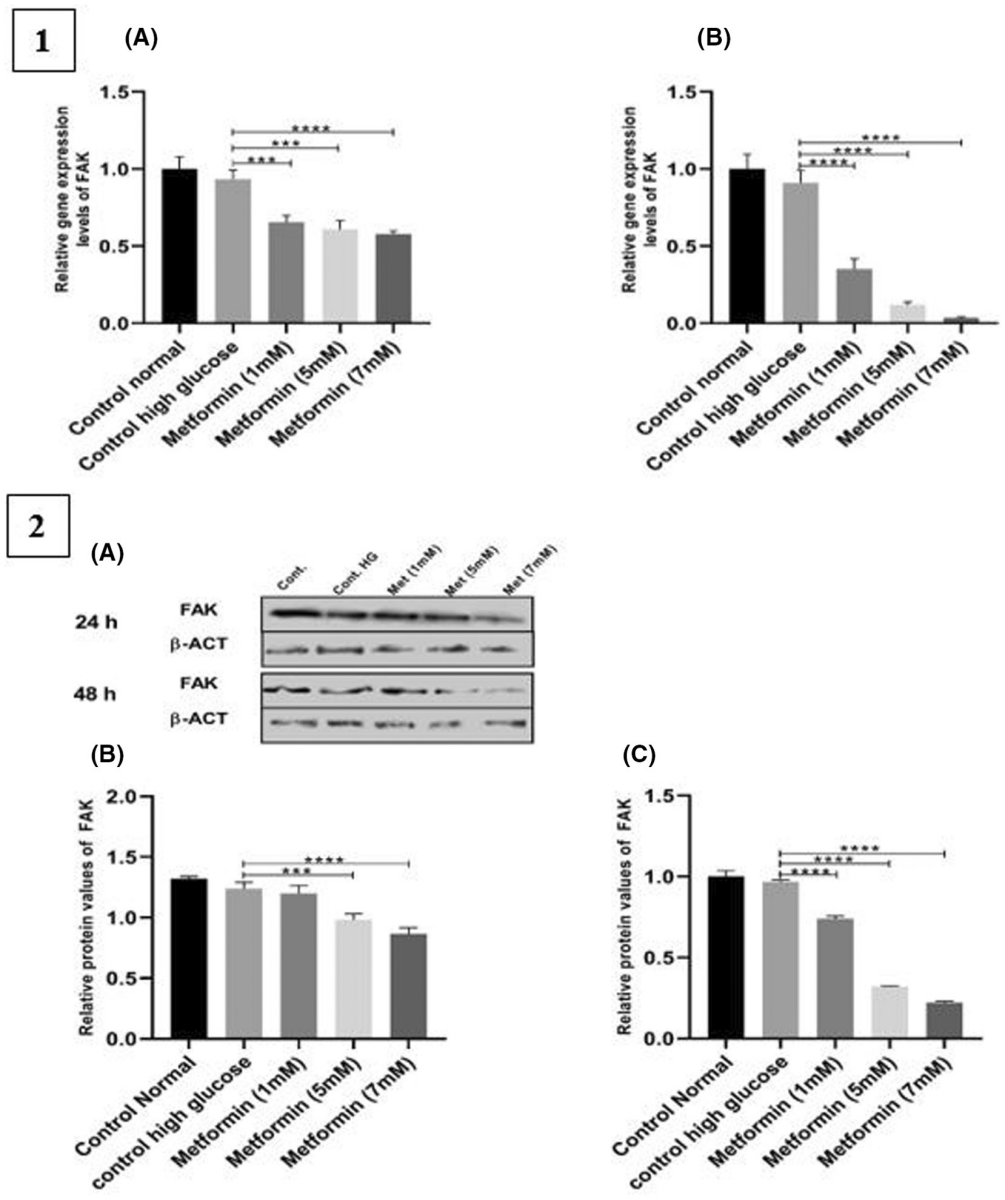
Gene and protein values of FAK. 1. FAK gene expression levels. VSMCs were treated with metformin (1, 5 and 7 mM) for 24 h and 48 h periods. (A) After 24 h of treatment, metformin significantly reduced the levels of FAK gene expression in the treated groups (control high glucose vs. metformin (1 mM) *p* = .0007; control high glucose vs. metformin (5 mM) *p* = .0002; control high glucose vs. metformin (7 mM) *p* < .0001)). (B) 48 h period, FAK gene expression levels decreased in all the metformin‐treated groups (control high glucose vs. metformin (1 mM) *p* < .0001; control high glucose vs. metformin (5 mM) *p* = .0001; control high glucose vs. metformin (7 mM) *p* < .0001)). 2. Protein values of FAK. (A) Western blot of pFAK protein. SMCs were treated with metformin doses (1, 5 and 7 mM) for (B) 24 h period (control high glucose vs. metformin (1 mM) *p* = .8361; control high glucose vs. metformin (5 mM) *p* = .0006; control high glucose vs. metformin (7 mM) *p* < .0001)) and (C) 48 h period (control high glucose vs. metformin (1 mM) *p* < .0001; control high glucose vs. metformin (5 mM) *p* < .0001; control high glucose vs. metformin (7 mM) *p* < .0001)). Data are repeated three times (*n* = 3) and are presented in mean ± SD. ***p < .001, ****p < .0001

The metformin significantly reduced the FAK gene expression levels in all the treated groups in both 24 h (*p* < .0008) and 48 h (*p* < .001) periods as compared to the control high glucose group (Figure 3,1A,1B).

### 3.3 pFAK protein values

The results showed that 5 and 7 mM metformin values significantly reduced the FAK values (*p* < .0008). However, metformin dose (1 mM) had no significant difference in the Fak protein at 24 h period (*p* > 0.8). Furthermore, the FAK protein values significantly decreased at 48 h after treatment with different doses of metformin (1, 5 and 7 Mm) (*p* < .0002) (Figure 3,2A–C).

### 3.4 Cell migration

**FIGURE 4:**
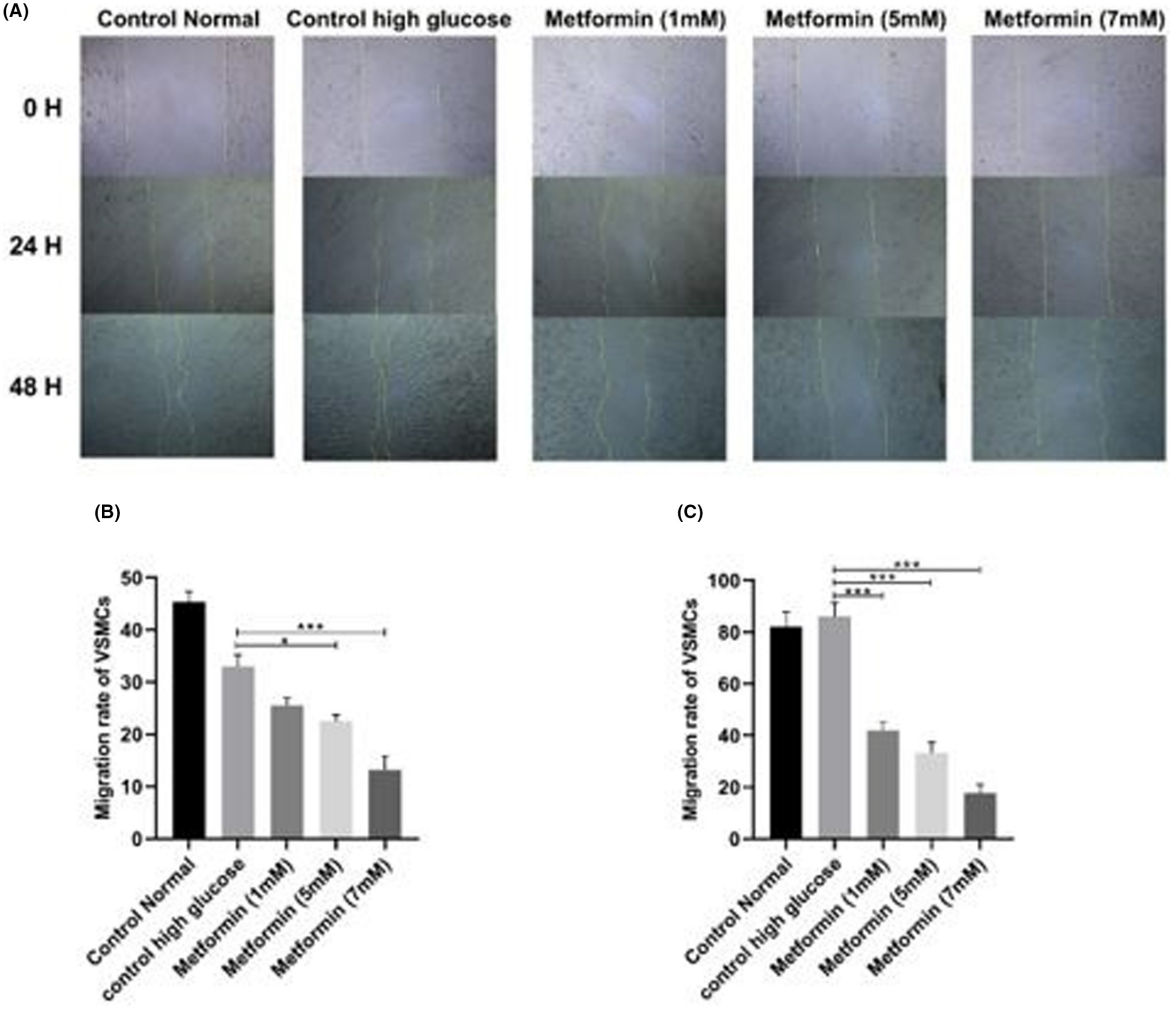
The effects of metformin on VSMCs migration. (A) Microscopic images of VSMC incubated with different doses (1, 5 and 7 mM) of metformin. Cellular migration was changed after 24 h and 48 h periods. (B) 24 h period (control high glucose vs. metformin *p* = .0645; control high glucose vs. metformin (5 Mm) *p* = .0166; control high glucose vs. metformin (7 Mm) *p* < .001)). (C) 48 h period (control high glucose vs. metformin *p* = .0009; control high glucose vs. metformin (5 Mm) *p* = .0004; control high glucose vs. metformin (7 Mm) *p* < .0001)). Data are repeated three times (*n* = 3) and are presented in mean ± SD. * *p* < .05, *** *p* < .001

As shown in Figure 4, the metformin values 5 and 7 mM significantly decreased the migration in 24 h period (*p* < .05). Additionally, at 48 h period, a significant decrease in cellular migration was observed in all the concentrations of metformin (1, 5 and 7 mM) (*p* < .001) (Figure 4).

## 4 DISCUSSION

It is well known that some cardiovascular complications such as myocardial infarction, heart failure, and micro and macrovascular events are more prevalent in diabetes.^1^, ^12^ Some studies showed that hyperglycemia is a paraclinical characteristic of diabetes that has been related to atherosclerosis.^4^, ^13^, ^14^ Atherosclerosis is a disorder in which atherogenic plaques are built within the heart's coronary artery walls, narrowing them and causing vessel stenosis. One of the most important features involved in atherosclerosis is the proliferation and migration of VSMCs in vessel subendothelial space.^15^, ^16^ The studies showed that in the high glucose conditions, growth‐promoting substances and cytokines activate the proliferation and migration of VSMCs in the intermediate layer of the vessel wall.^17^, ^18^ The FAK is known as one central protein of many signalling pathways involved in cellular proliferation and migration events.^19^ Its phosphorylated form plays a crucial role in cell signalling transduction. Some studies showed that FAK affects VSMC migration via the RhoA/ROCK1 signalling pathway.^20^, ^21^ FAK also is activated by EGFR and integrin receptors through cytokine‐stimulated pathways.^22^ On the other hand, studies have shown that metformin has a glucose‐lowering effect and affects some molecular pathways. Metformin is also suggested to have anti‐inflammatory properties.^5^, ^23^ Other studies reported that metformin inhibits AMPK‐mediated VSMC proliferation and migration.^24^ Moreover, metformin has also been shown to inhibit vascular calcification via the AMPK/eNOS/NO signalling pathway, suggesting that it may have therapeutic potential for vascular calcification in type 2 diabetic complications.^25^ In this study, the effects of metformin were investigated on the FAK protein values and its gene expression levels, and on the proliferation and migration of vascular smooth muscle cells in high glucose conditions. The results showed that metformin reduces the FAK gene expression levels and protein values in VSMCs in a dose‐dependent manner. Metformin also decreased cellular proliferation, so that the values above 16 mM of metformin affect effectively the VSMC viability. The results also showed that metformin decreases the VSMC migration according to the FAK protein changes, so that these data suggested that metformin may retard atherosclerosis in diabetes. In contrast with some studies,^26^, ^27^ the high glucose conditions did not affect cellular proliferation and migration. It was in agreement with the previous report that the cell viability and migration do not correlate with glucose values up to 50 mM.^28^ It was proposed that the changes of VSMC phenotype in high glucose conditions are related to molecular intermediates produced by other cells,^4^ so that it was proposed to study in animal models.

## 5 CONCLUSION

The results showed that metformin suppresses the VSMC proliferation and migration in high glucose conditions. However, the results may improve by focusing on the molecular mechanisms related to downstream genes of FAK‐related signalling pathways.

## AUTHOR CONTRIBUTIONS

**Ali A Soleimani:** Investigation (supporting). **Ghasem Ghasmpour:** Investigation (supporting). **Asghar Mohammadi:** Investigation (supporting). **Masoomeh Gholizadeh:** Investigation (supporting). **Borhan R Abkenar:** Investigation (supporting).

## CONFLICT OF INTERESTS

None declared.

## Data Availability

The data were presented on the request.
